# COVID-19 influences on US recreational angler behavior

**DOI:** 10.1371/journal.pone.0254652

**Published:** 2021-08-18

**Authors:** Stephen R. Midway, Abigail J. Lynch, Brandon K. Peoples, Michael Dance, Rex Caffey

**Affiliations:** 1 Department of Oceanography and Coastal Sciences, Louisiana State University, Baton Rouge, Louisiana, United States of America; 2 US Geological Survey, National Climate Adaptation Science Center, Reston, Virginia, United States of America; 3 Department of Forestry and Environmental Conservation, Clemson University, Clemson, South Carolina, United States of America; 4 Department of Agricultural Economics, Louisiana State University, Baton Rouge, Louisiana, United States of America; Swansea University, UNITED KINGDOM

## Abstract

Recreational angling in the United States (US) is largely a personal hobby that scales up to a multibillion-dollar economic activity. Given dramatic changes to personal decisions and behaviors resulting from the COVID-19 pandemic, we surveyed recreational anglers across the US to understand how the pandemic may have affected their fishing motivations and subsequent activities. Nearly a quarter million anglers from 10 US states were invited to participate in the survey, and almost 18,000 responded. Anglers reported numerous effects of the pandemic, including fishing access restrictions. Despite these barriers, we found that the amount of fishing in the spring of 2020 was significantly greater—by about 0.2 trips per angler—than in non-pandemic springs. Increased fishing is likely associated with our result that most respondents considered recreational angling to be a COVID-19 safe activity. Nearly a third of anglers reported changing their motivation for fishing during the pandemic, with stress relief being more popular during the pandemic than before. Driven partly by the perceived safety of s*ocial fishtancing*, recreational angling remained a popular activity for many US anglers during spring 2020.

## Introduction

Recreational angling is fishing with the principal motivation of leisure, and has important social, economic, and conservation values [1]. In the United States (US) alone, more than 49 million people identify as recreational anglers [2], making recreational angling second only to jogging in terms of popular outdoor activities [3]. In addition to being highly popular, recreational fishing is also an important component of the US economy. In 2016 alone, recreational angling expenditures totaled nearly US$50 billion and more than 800,000 livelihoods were enhanced by recreational fishing [4]. These expenditures generated $6.5 billion in state/local tax revenues and $9.4 billion in federal tax revenues. Moreover, excise taxes from recreational angling expenditures are the primary funding source for many conservation efforts across the US [5]. Consequently, management agencies are deeply vested in understanding how external influences can drive the behaviors and decisions of recreational anglers as they impact the magnitude and scope of management initiatives.

The reasons anglers go fishing are as diverse as the people engaged in fishing activities. Common recreational angler motivations can be psychological (e.g., relaxing, getting away), social (i.e., interacting with other anglers), competitive (e.g., fishing tournaments), or may simply reflect a desire to experience nature [6]. Several other reasons may exist, such as fishing for food [7, 8] or developing skills, but these are typically lower in importance in the US and may vary in importance based on demographics. Most recreational anglers have multiple motivations for fishing, and understanding the relative ranks of these motivators is important for managing recreational fisheries [6]. Moreover, recreational angler motivations are not static, and changes in motivations affect participation in a fishery and subsequent fishing license sales—another important revenue stream for fisheries conservation efforts [9]. Because angler motivation and participation are often changing, even in the face of relatively constant conditions, it is very important to document changes to angler motivation as it responds to large-scale environmental or societal changes.

In early 2020, the world experienced the effects of a global pandemic from the SARS-CoV-2 virus causing COVID-19 (hereafter, COVID-19). To prevent spread of the virus, many nations and jurisdictions implemented strict lockdown orders to limit movement by individuals. These restrictions had a profound effect on the global socioeconomic landscape, causing billions of workers to temporarily or permanently lose employment or wages, work from home, or continue work as normal and risk infection; this restructuring caused dramatic shifts in consumer habits that are likely to be long-lasting [10]. Several recent reports examining the effects of COVID-19 on fisheries [e.g., 11–13] suggest that reduced consumer pressure has negatively affected markets [14], but has potential for positive effects on fishery resources [12, 13]. Yet while the effects of COVID-19 restrictions have been studied for other forms of outdoor recreation [15, 16], effects on recreational fishing participation and motivation remain mostly unknown. Moreover, differences in COVID-19 restriction policies among nations, states, or provinces can cause heterogeneous effects on fishing in different regions [13, 17–19]. For example, strict lockdown policies in urban areas have reduced opportunities for outdoor recreation in urban areas, while outdoor recreationists in rural areas have been less affected [20]. Quantifying effects of COVID-19 restrictions on US recreational fishing and angler motivation provides a first step toward understanding the social and economic impact of the pandemic on this valuable industry.

The unprecedented nature of the current COVID-19 pandemic is a significant unknown in terms of effects on angler motivation and participation. On one hand, lockdowns and travel restrictions may limit participation in recreational fishing that involves long-distance and potential out-of-country travel, as has been observed in other forms of ecotourism [18, 21]. On the other hand, much recreational fishing in the US is done fairly close to home [22, 23], and COVID-19 restrictions have caused outdoor recreationists to drive shorter distances for recreational opportunities [24]. Moreover, recreational fishing is fairly inexpensive if anglers already possess the necessary equipment and reside in close proximity to fishing access points. Accordingly, it is also possible that the pandemic has affected recreational fishing less than other forms of outdoor recreation. With very few studies examining the impacts of COVID-19 on fisheries [see 13], management agencies should consider a wide range of hypothetical outcomes. For example, the shelter-in-place orders that extended across numerous US states could be expected to decrease angling opportunities as anglers were faced with the numerous burdens (e.g., health and economic) that accompanied the pandemic. Conversely, increased time away from work and the desire for socially distanced activities could lead to an increase in angling [e.g., *social fishtancing*; 25].

This study was carried out during summer 2020 to examine the impacts of the pandemic on fishing habits and behaviors of US anglers. Our objectives were to directly survey salt- and freshwater anglers to provide an initial understanding of the effects of COVID-19 on angling behavior, whether angling effort differed from recent (non-pandemic) years, whether the reasons for going fishing were changed, and whether any angling access was limited. The intention for this study was to report on preliminary findings and descriptions on a topic that is entirely new and emerging for the world. The purpose of this study was to simply document how anglers are being affected by the pandemic and other entirely unknown responses. Our results now can inform future work and parameterize future statistical models on this topic.

## Materials and methods

### Survey design

In the spring and summer of 2020, we developed a survey to collect information on US recreational angler attitudes and behaviors during the early months (March–June) of the COVID-19 pandemic in the US. We designed the survey for private recreational salt- and freshwater anglers, and not recreational-for-hire or any other recreational group. The survey (which can be found in its entirety in S1 Appendix) included 20 questions that were designed to collect information on: 1) the overall and primary effects of the pandemic on anglers, 2) the change in number of fishing trips between a typical spring and spring 2020, 3) reasons anglers fished before and during the pandemic, 4) whether fishing access was changed during the pandemic, and 5) overall perceptions of safety associated with fishing (i.e., in relation to the possibility of contracting COVID-19). Information about respondent location (US state) and demographics were also collected; however, all responses were anonymized.

Every survey question was optional so that there were no barriers to completing the survey if a respondent did not want to complete one or more questions. Throughout the survey, use of the word *typical*, as in a typical spring, was defined as non-pandemic (prior to 2020). The survey also used the term *fishing trip*, although we did not define it because the diversity of angler habits and fisheries creates complexity around defining a *trip* for a broad population, such that our inability to predict everyone’s definition of a trip could unintentionally exclude them from the survey. While it may not be ideal to leave a *fishing trip* undefined, the goal was for anglers to tell us whether they fished more or less during the pandemic. Our determination was that the term *fishing* alone would likely work. However, the term *fishing trip* has a suggestion of a unit of fishing, and for certain questions in the survey we were attempting to quantify the amount of fishing and having some generalized unit would help that effort. A fishing trip can certainly be a variable unit, but the term is commonly used within fishing communities and at least permit us to draw inferences about increases or decreases in units of fishing. We expected that by avoiding technical units and other complexities that could confuse respondents, we might get a better sense of the overall answer to whether anglers fished more or less during the pandemic. All survey actions—survey design, angler participation, and communication—were carried out through the survey software Qualtrics with Louisiana State University Institutional Review Board approval (IRB# E12321).

### Recruitment of states

**Fig 1:**
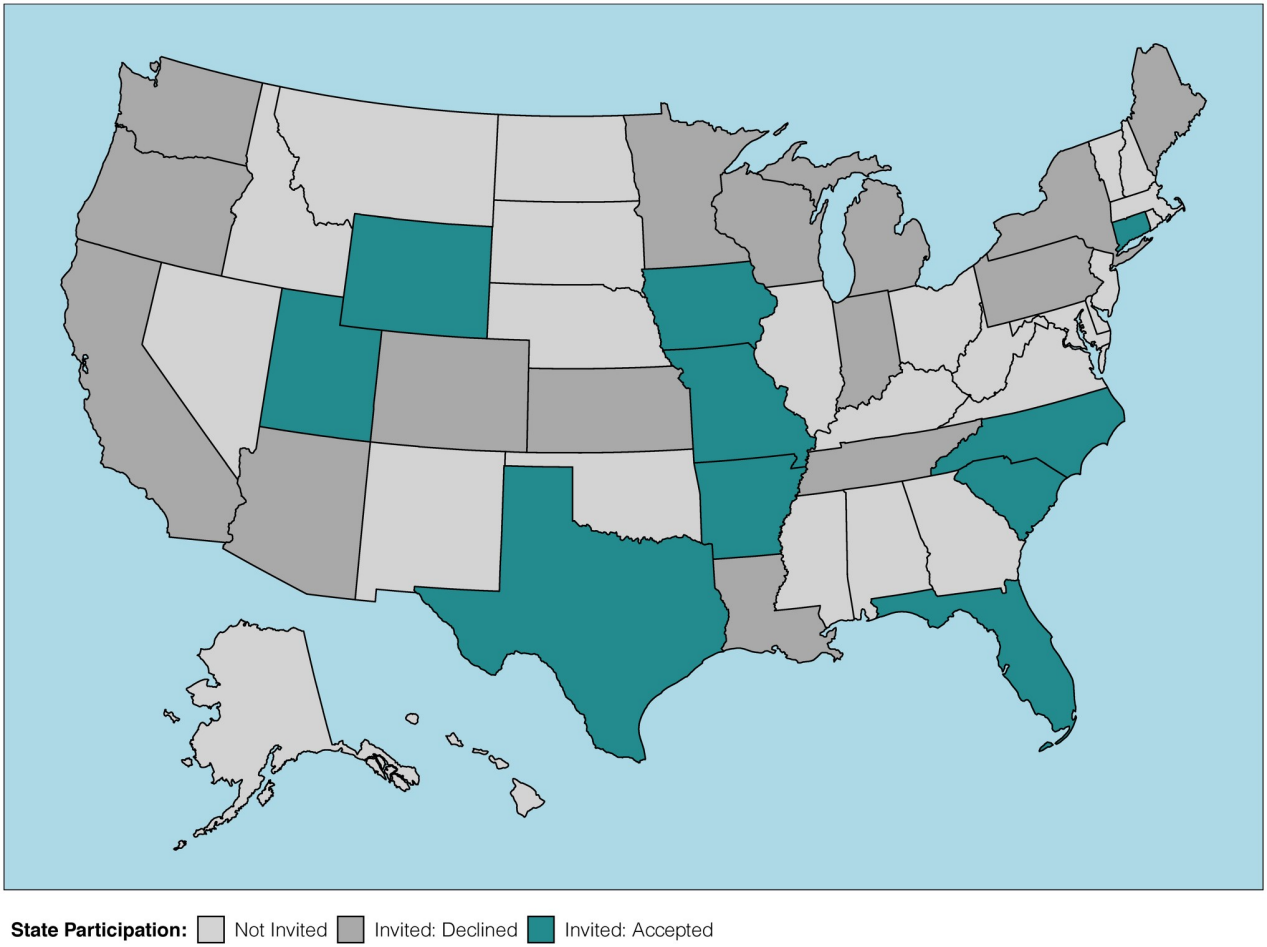
Map of participating US states. US states are shaded to indicate whether a state was invited to participate in the survey, and if invited, whether they declined or accepted.

We wanted the survey to be taken in the summer of 2020 while spring attitudes and behaviors were still in recent memory. We sought to carry out a probabilistic study design by inviting a known number of recreational anglers to participate in the survey—as opposed to something like an open-access non-random (public) survey that would not allow estimation of response rate and likely include non-licensed anglers, among other concerns. To contact a known number of licensed anglers, we reached out to 25 US state fishery and wildlife agencies with an invitation to participate by sharing email contact information for a random sample of licensed resident anglers. Because many anglers rely on public access to fish, we wanted to analyze changes to access at the state level (i.e., opening and closing public access to fishing would most likely be influenced by state governments). Of the 25 states invited to participate, 15 states declined due to various reasons, including inability to share license holder emails (e.g., legal prohibitions), concerns of license holder survey fatigue from existing agency questionnaires, inability for agency personnel to devote time to the request, and non-response. Ultimately, 10 states participated: Arkansas, Connecticut, Florida, Iowa, Missouri, North Carolina, South Carolina, Texas, Utah, and Wyoming (Fig 1). All state participation occurred with appropriate legal documentation in place (e.g., memorandum of understanding, non-disclosure agreement), as outlined and approved by each state.

We targeted 1,000 responses per state, which is approximately a sample size that corresponds to 3% sampling error [26]. Given this target, we assumed a 5% response rate. Although this assumed response rate is low, the large, external nature of the survey along with the survey taking place during a pandemic, suggested the need for a conservative estimate of participation. Our target of 1,000 responses and estimated 5% response rate led us to request 20,000 email addresses from participating state agencies. Ultimately, states provided a variable number of email addresses ranging from 852 to 985,204 (Table 1), and no states were excluded from participating based on the sample size they provided. Three states provided less than 20,000 email addresses, because they were either limited by the total number of email addresses they had available (e.g., Wyoming, where licensed anglers are not required to provide an email address) or limited based on other reasons (e.g., survey fatigue among licensed anglers). For states that provided ≥20,000 email addresses, we randomly sampled between 20,000–25,000 email addresses, with the exception of Florida and South Carolina, where 50,000 email addresses were used. (Florida and South Carolina provided the most email addresses to the survey and both are states with freshwater and saltwater anglers, which was a reason we increased the sample, although we did not seek to investigate habitat-based effects because the survey was not designed for that reason).

**Table 1:** States, number of anglers invited to participate in the survey, number of surveys taken, and response rate for a COVID-19 survey.

State	Anglers Invited	Surveys Taken	Response Rate
Arkansas	23,403	1,834	7.8%
Connecticut	19,163	1,584	8.3%
Florida	47,291	3,022	6.4%
Iowa	23,759	2,093	8.8%
Missouri	23,952	1,718	7.2%
North Carolina	24,701	2,073	8.4%
South Carolina	42,607	3,847	9.0%
Texas	13,100	1,171	8.9%
Utah	5,332	545	10.2%
Wyoming	753	96	12.7%
**Overall**	**224,061**	**17,983**	**8.0%**

(The number of anglers contacted excluded any duplicate emails and bounced emails).

### Survey period

On July 23, 2020, the 20-question survey was distributed via email to 241,352 licensed recreational anglers from 10 US states. Of the total number of emails provided, a small percent was invalid (e.g., email addresses that rejected or bounced or were duplicate email addresses provided by the state agency) resulting in a total of *n* = 224,061 email requests reaching potential survey participants (Table 1). On August 4, 2020 a single reminder email was distributed only to those who had not started or completed the survey. Subsequent reminders were not sent because target samples sizes were approached, and we wanted to minimize undue contact. The survey was closed on August 14, 2020.

## Results and discussion

A total of 17,983 surveys were completed for an overall 8.0% participation rate, which ranged from 6.4–12.7% among states (Table 1). Although 17,983 surveys were taken, our usable sample size was 16,919 surveys due to 1,064 surveys that were removed from the data because the initial question asking for consent was not permitted or left blank (i.e., we did not assume consent unless the question response was *Yes*). Due to the option for survey respondents to skip questions without penalty, not all completed surveys had responses for every question. Despite the option to skip questions, however, most survey respondents did not skip many questions and, as such, response rates to individual questions are generally high (>90%). Generally, state of residence was not a strong factor underlying changes in motivations and behaviors that we observed, and as such, we often pooled anglers across states and evaluated survey response based on other factors. Pooling was done by simply combining raw responses among states for analyses; no weighing, averaging, or other metrics were necessary.

### Effects of the pandemic on anglers and their effort

**Fig 2:**
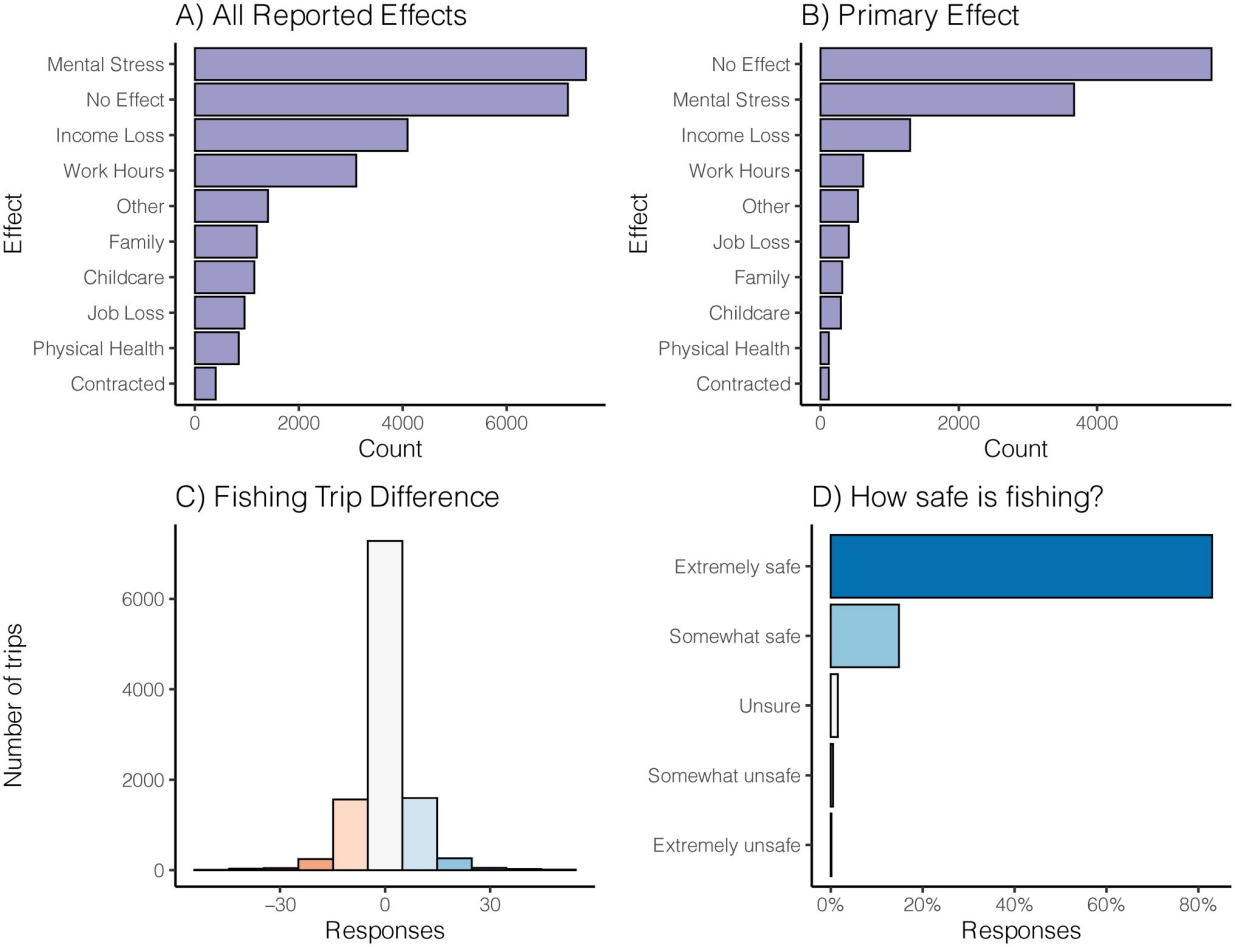
Pandemic effects on anglers, changes in fishing trips, and perceived safety of fishing. All reported pandemic effects (A) and the single primary pandemic effect (B) on anglers from 10 US states (see Fig 1 or Table 1 for states). Panel C shows the numeric difference in fishing estimated fishing trips during a typical spring and that of the 2020 pandemic spring. Panel D displays the perceptions of how safe recreational fishing is. (Note that data for all four panels were collected and analyzed at the state level but were aggregated here due to no strong state effects).

A total of 10 pandemic effects (including *Other* and *No Effect*) were reported from 14,422 anglers with *Mental Stress* and *No Effect* being the most common (when ranked by primary effect [i.e., including either the top ranked reported effect when more than one effect was reported, or the single effect reported when only one effect was reported]; Fig 2A). As with the overall effects, *No Effect* and *Mental Stress* were the top reported effects; however, *No Effect* was much more common than *Mental Stress* (Fig 2B).

**Fig 3:**
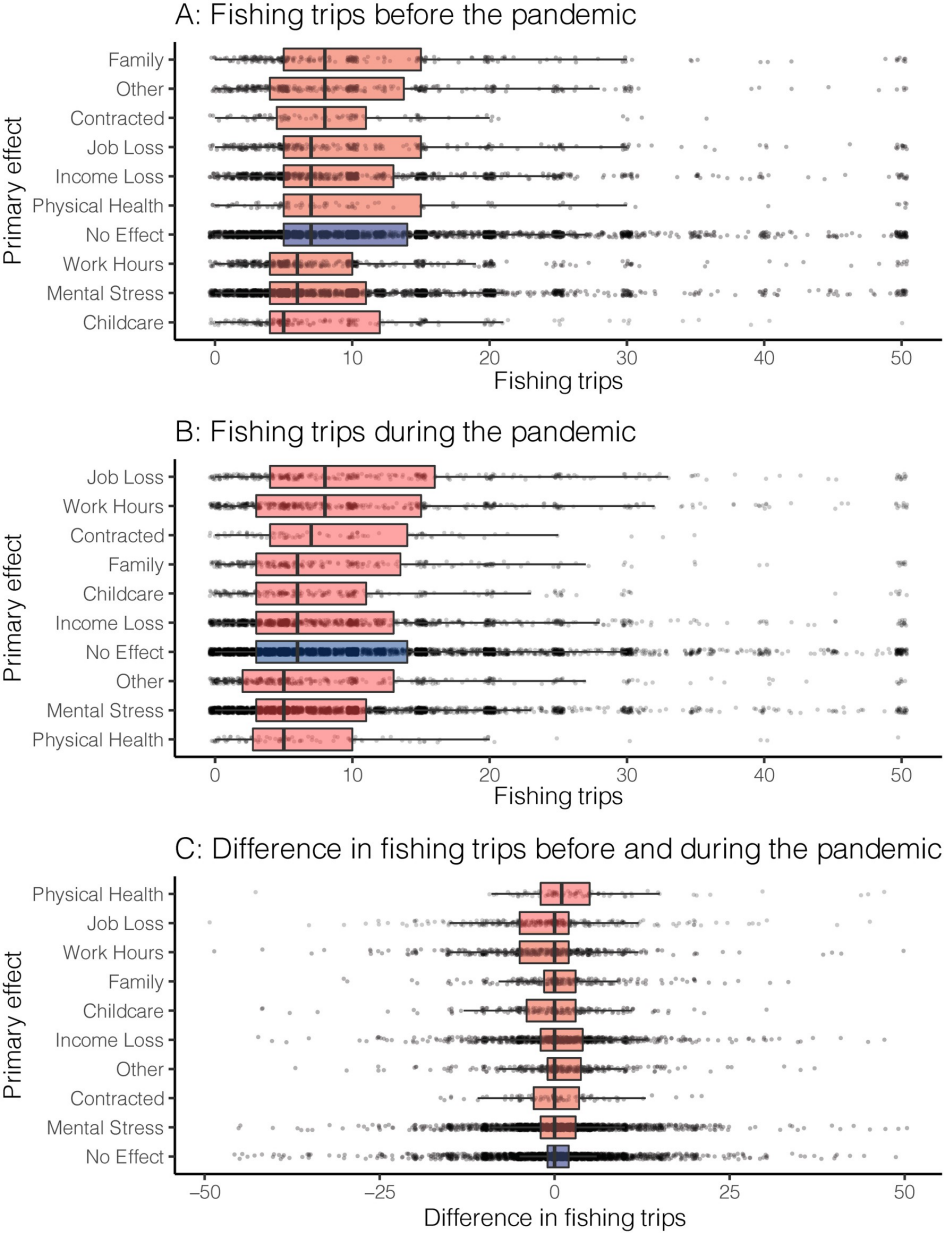
Boxplots of fishing trips grouped by primary effect of the pandemic. Fishing trips before (panel A) and during (panel B) the pandemic, along with the difference in fishing trips (panel C) are broken down by angler’s primary reported effect. Individual responses are shown in jittered gray dots (which overlap to appear black), and boxplots are shaded red or blue to simply distinguish between primary effects (red) and no effect (blue). Boxplot are ordered by the mean number of trips, with the most trips in the top box and least trips in the bottom box.

Respondents fished more during the pandemic, compared to the previous year based on the number of self-reported fishing trips between a typical spring and the pandemic spring of 2020. A *Z*-test revealed that the increase in trips was significantly different from 0 (95% confidence interval 0.12–0.38 trips). However, the mean number of trips during the pandemic spring was only 0.25 trips greater than a typical spring (with SD = 7.5; Fig 2C). In order to look more closely at changes in fishing trips, we also analyzed and compared the individual primary effects of the pandemic on fishing trips. To do this, we took all reported primary effects and ran three ANOVA models: one model comparing the number of fishing trips before the pandemic (data presented in Fig 3A), one model for comparing the number of fishing trips during the pandemic (data presented in Fig 3B), and one model comparing the difference in fishing trips before and during the pandemic (data presented in Fig 3C). Each of the three ANOVAs was significant (*p* < 0.01), indicating that the number of fishing trips differed by primary effect of the pandemic on anglers. Tukey HSD post hoc comparisons [27] were run on all three ANOVAs to determine which pairwise comparisons differed (see S1 Table for complete list of significant pair-wise comparisons). For fishing trips before the pandemic, mental stress or job loss was present in all six of the significant pairwise comparisons. When looking at the significant pairwise comparisons of primary effects on fishing trips during the pandemic, job loss was in six of eight comparisons, and mental health in the remaining two. Finally, there were 15 significant pairwise comparisons for the difference in fishing trips before and during the pandemic. The primary effects of work hours and physical health were the most common, with each represented six times.

Perhaps underlying this increase for all anglers was the fact that fishing was also considered to be *extremely safe* or *somewhat safe* by the vast majority (83%) of respondents, while very few anglers (1%) considered fishing to be *unsafe* (Fig 2D). Respondents also considered fishing to be a psychologically beneficial activity that was robust to the mental and physical challenges of a pandemic. Because recreational fishing is typically done alone or with small groups of trusted friends, the inherent safety of recreational angling has recently become colloquialized with the term *social fishtancing* in popular media such as magazines, podcasts, and social networking platforms. *Social fishtancing* implies that the recommended safe practice of social distancing, or maintaining about 6 feet of separation between yourself and others, is practically a requirement for recreational fishing. Accordingly, our results corroborate general angler viewpoints that recreational fishing can be done safely and responsibly during the pandemic.

### Why anglers fished

During the COVID-19 pandemic, many human behaviors changed as a result of numerous motivators and stressors. US recreational anglers, perhaps, may reasonably reflect the overall US population in terms of how they were affected by the pandemic. Anglers reported varied negative effects, which were likely also experienced by the general public. Pandemics *writ large* are well known for causing negative health effects (including mortality), income loss, and behavioral changes, among other effects [28]. The COVID-19 pandemic is likely to exhibit all the negative effects associated with general pandemics [29], but perhaps even more due to scope and scale of COVID-19 cases around the world.

**Fig 4:**
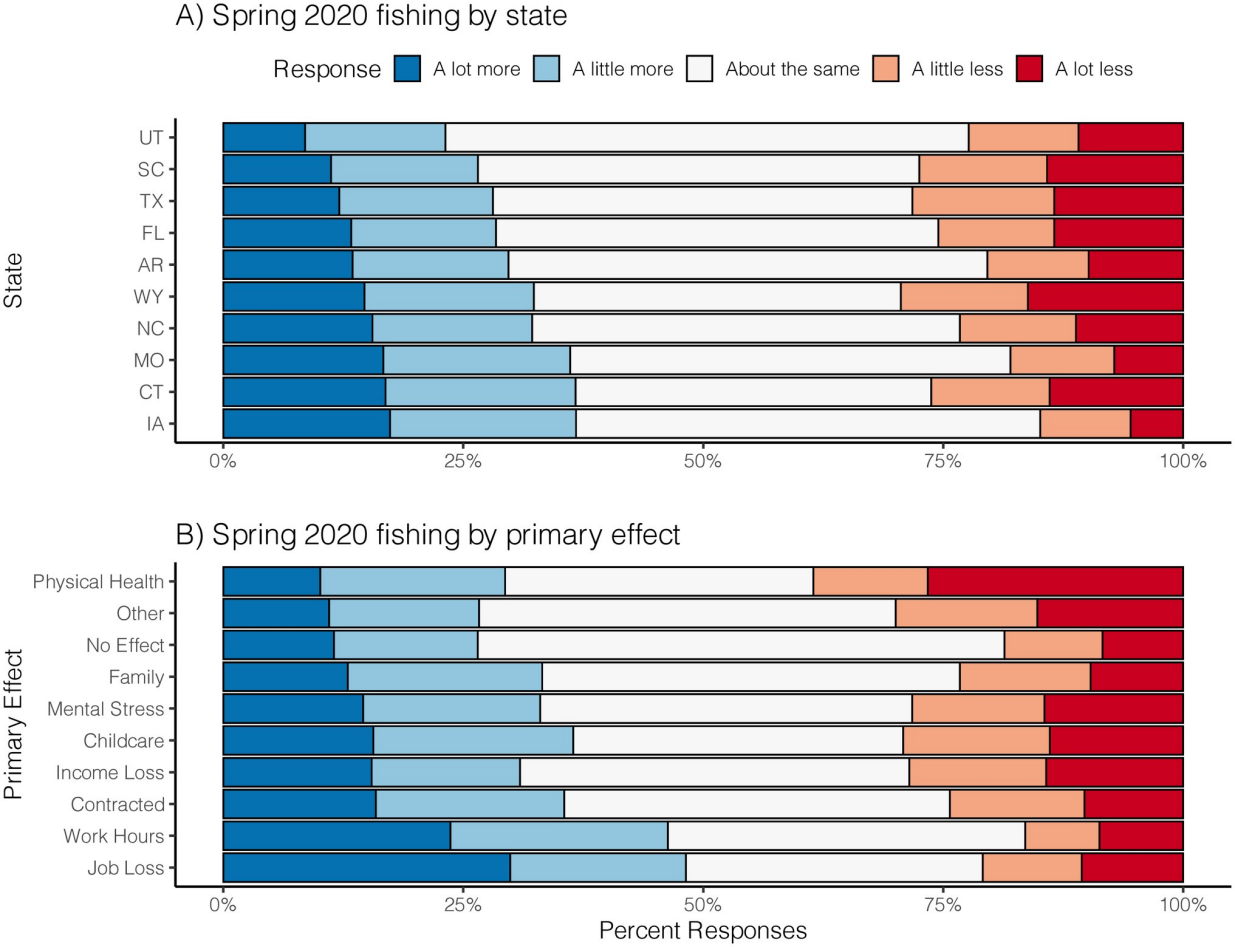
Breakdown of self-reported fishing in spring 2020 (during pandemic) compared to a typical spring. Note that the data are the same responses, but grouped by state in panel A and grouped by primary pandemic effect panel B.

Breaking down spring 2020 fishing by primary effect of the pandemic provided more contrast in differences compared to the state breakdown (Fig 4A). Most primary effects were associated with 30–35% of respondents reporting they fished more than in a typical spring, yet nearly 50% of respondents with lost work hours or lost jobs reported fishing more (Fig 4B). In contrast, 20–30% of respondents for most primary effects reported less fishing compared to a typical year, with the exception of those reporting physical health as a primary effect.

**Fig 5:**
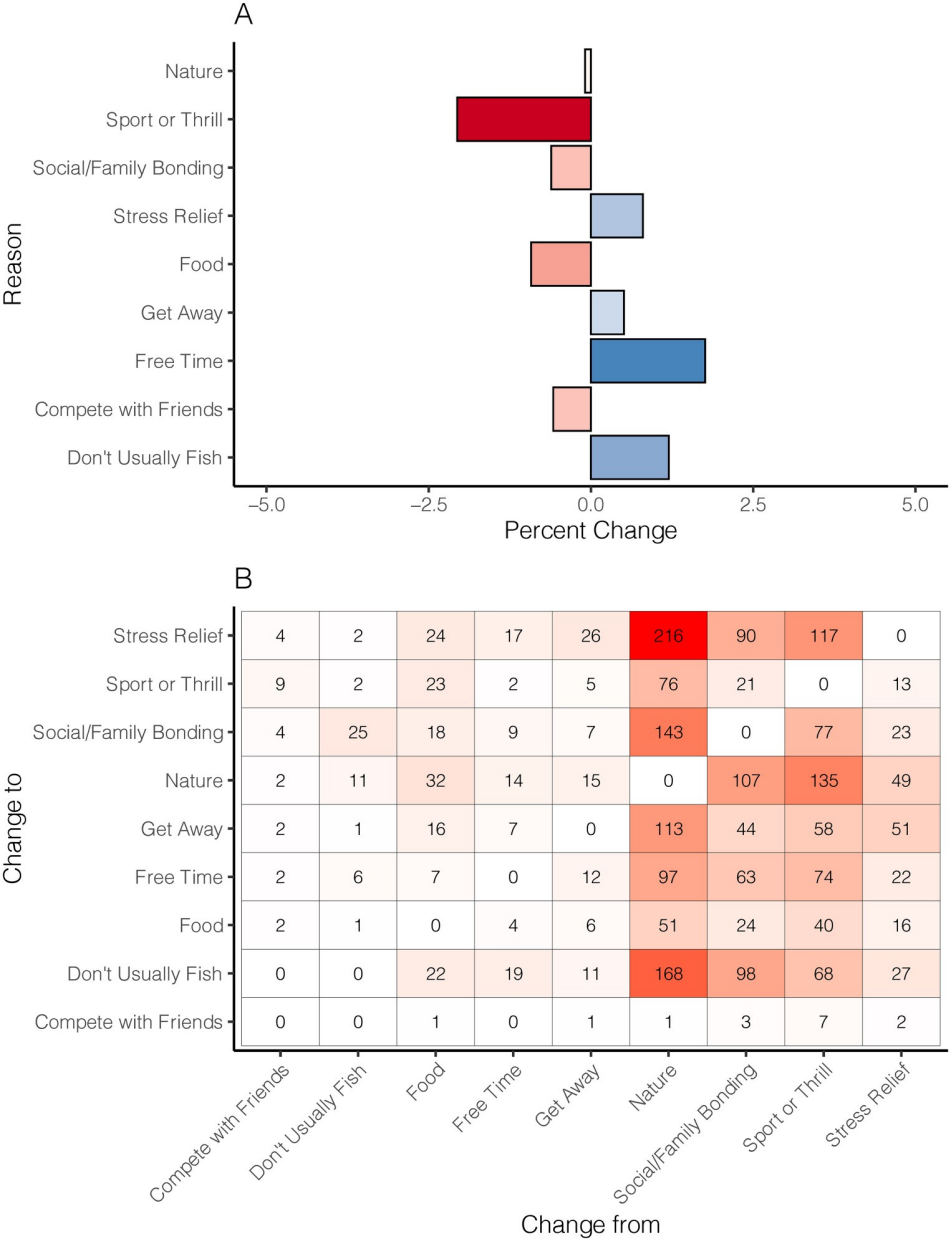
Changes in reasons anglers fished. Panel A—Percent difference in the total reported reasons anglers fished before the pandemic compared to during the pandemic (A). Negative (red) percentages represent a reason that declined during the pandemic (compared to before the pandemic) and positive (blue) percentages represent a reason that increased during the pandemic (compared to before the pandemic). Panel B—Heat map of the combinations of changes of primary reasons for fishing from before the pandemic (*x*-axis) to during the pandemic (*y*-axis). Darker values (reds) indicate larger changes and raw values are included for each combination. Overall, 65% of respondents reported changing their primary reason to fish.

Changes in reasons that anglers fished (i.e., motivators) showed two main findings. The first finding was that the overall proportion of motivators was not substantially different before or during the pandemic. Because the reported number of motivators before and during the pandemic was not the same, we compared percentages as a way to standardize across time. The mean magnitude of change among motivators was only 0.9%, with the largest decrease associated with the *Sport or Thrill* motivator and the largest increase associated with *Free Time* resulting from the pandemic (Fig 5A). Despite the relatively minor changes in proportions of before and after motivation, our second main finding was that 35% of anglers reported changing their primary motivator for fishing during the pandemic. The motivator with the greatest increase was *Stress Relief* (Fig 5B); however, *Social/Family bonding* and *Nature* were also popular pandemic motivators. These changing motivators stand to reason because, while they are generally popular motivations to fish, these three reasons are also coherent with the perceived safety of fishing and connote psychological benefits. Interestingly, several respondents changed their responses to *Don’t usually fish* during the pandemic, suggesting there was some segment of anglers that previously did fish (for different reasons), but did not during the spring and summer of 2020. Collectively, our findings suggest that (with the possible exception of limiting access) anglers are going to go fishing regardless of local, regional, or national conditions and the reasons motivating the fishing trip may be the only substantial change in this behavior. Indeed, hobbies and outdoor activities were associated with lower levels of depressive symptoms in Spanish adults during the spring 2020 COVID-19 lockdown [30], and the enjoyment of fishing favorably compares to most hobbies and outdoor activities.

Among anglers who changed how much they fished, changes appeared to be associated predominately with how individuals were affected by the pandemic. Specifically, nearly 50% of anglers reporting job loss or reduced work hours fished more during the pandemic than a typical spring. This may be because with less time committed to work, anglers could increase their engagement in a safe, low-cost, and mentally beneficial activity like fishing. It is worth noting, however, that fishing trips increased even more for anglers not experiencing job loss or reduced work hours. Interestingly, loss of childcare was also associated with an increase in fishing during the pandemic. Our study did not ask whether children were included in fishing trips, but it is very possible that the pandemic resulted in an increase in child and youth angling by parents who were seeking fun and safe outdoor activities for kids who did not have school and care facilities open. Unsurprisingly, anglers reporting physical health effects of the pandemic had the greatest reductions in fishing, with no other effects strongly associated with fishing reductions.

### Changes to angling access

**Fig 6:**
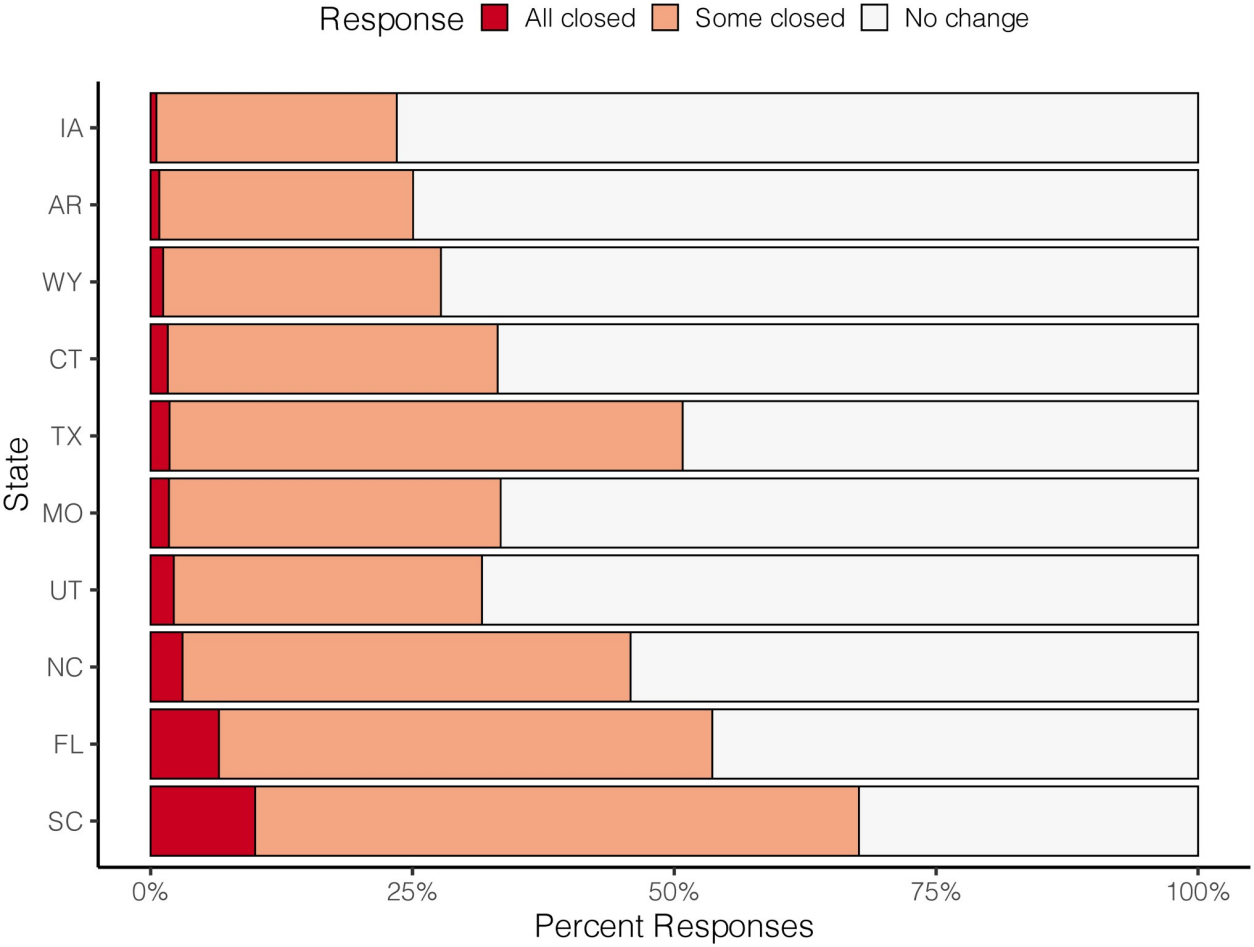
Angler-reported changes to fishing access grouped by state.

We found relatively little differences across states in terms of complete loss of access (*All closed*); 10% or less of most respondents across all states reported having all their access closed (Fig 6). However, proportions of state respondents reporting loss of some access (*Some closed*) were highly variable. Only 25% of anglers in Iowa and Arkansas reported some loss of access, whereas >50% of respondents from Texas, Florida, and South Carolina reported some loss of access.

This study was designed to include different states from different regions of the US, with the thinking that possible state-to-state variability in the severity of COVID-19 cases and state-specific pandemic responses (e.g., lockdown, travel restrictions) might lead to angling behaviors that were very state-specific. State-specific responses, by-and-large, did not correlated, with the exception of limited access. Because so much fishing access in the US is public and some states did close or limit fishing access in the spring of 2020, it stands to reason that we found differences among states when it came to access. Specifically, respondents reported between 25–75% of their fishing access being fully or partially closed. However, reported negative effects from the pandemic were largely consistent among states while reports of increased or decreased effort showed greater differences associated with particular negative effects. Although individual US states license anglers, control most public access, and have local and regional fisheries, our overall results point to a more general response among anglers that was not apparently driven by angler location.

### Study limitations

There are other data sources that could address the questions we asked. State agency data from creel surveys and license sales, for instance, might be another way to address the topics we explored. In some ways, creel surveys and license sales may be more reliable than angler-reported information, which by its nature relies on angler memories and a desire to provide honest feedback in an anonymous survey [e.g., 31]. However, creel surveys and license sale data may also be difficult to interpret. For example, restrictions prevented some state management agencies from running creel surveys during part of the pandemic, causing key gaps in the data. Moreover, if quality creel survey data do exist, there are still challenges with obtaining the data in a timely manner (i.e., some states do not release data until months after collection, input, and archiving) and standardizing creel data among states [32]. License sales also pose challenges because states have different timelines for license expiration that may not capture changes to sales within a single season. Common models for annual licensing periods include from the day of purchase, over a calendar year, or over a fiscal year, among others. Such differences in timing may not completely obscure an increase or decrease in spring license sales; however, the license sale dynamics inherent to each timeline would need to be considered as they would likely make among state comparisons challenging.

Response rate and non-response bias are concerns common to most surveys. Although our response rates would generally be considered low (6–13%), note that there is no universal threshold for response rate and that metrics like margin of error and non-response bias can be more important when determining survey quality [26]. In fact, we expected a response rate around 5%, given the fact that this was an internet-based, external survey (i.e., large, public group) and both those factors are known to reduce response rates [33]. We also expected that the numerous stresses created by the pandemic (the same effects we sought to quantify) might only depress the responses as a population of people experiencing hardship might be less likely to donate their time and information than they would in other situations. Regardless, response rate alone is not necessarily a good predictor of response quality; for example, a response rate of 10–20% with a low margin of error could provide good information, while a survey with a high response rate may not be immune to non-response bias or other challenges.

Non-response bias is a larger concern for our study given there are several possible sources of non-response bias we could have encountered. Biases associated with identifying the wrong audience (i.e., coverage error) were likely not a factor as our survey participants were all licensed anglers with contact information provided by individual states in which anglers were licensed. We also recognize there may be biases with our web-based survey mode. However, an estimated 90% of Americans use the internet [34] and the fact that our survey was based on angler-provided email addresses strongly suggests they have internet access. Additionally, the web-based survey mode we selected was critical for inviting the large number of anglers needed for the survey, particularly considering pandemic restrictions for in-person approaches.

Two other possible sources of non-response bias might be more likely to have occurred in our survey. First, although all questions were optional and survey responses were anonymized, we did ask what could be perceived as sensitive information about the health of respondents. It is plausible that some anglers contracted COVID-19 or otherwise were so negatively affected by the pandemic that they did not want to relive or share their experiences in a survey. Another possible source of non-response bias is avidity, which is a common bias in angling surveys [35, 36]. An avidity bias can occur when more avid anglers have greater relative representation than less avid anglers. In other words, an active angler may be more likely to be interested in and complete a survey, while a less avid angler may have less interest or self-exclude based on the incorrect assumption that they do not fish enough to hold relevant opinions. Although the best way to test for non-response bias is to conduct a separate follow-up survey of those who did not respond the first time, and then to compare the responses between groups, we were only permitted to contact anglers for a single survey (to minimize survey fatigue). Given this limitation, a formal non-response analysis was not possible. Similarly, it would have been ideal to conduct a survey before the pandemic and then compare those responses to the survey presented in this study; however, the pandemic was not anticipated, and therefore a pre-pandemic survey could not be planned. Furthermore, the pandemic has ushered in new terms (e.g., social distancing) that were uncommon before the pandemic, and such a pre-pandemic survey would have had numerous challenges. This unfortunate lack of pre-pandemic baseline data highlights the importance of routine monitoring of angler perceptions to detect changes through time and enable more adaptive management approaches.

Despite challenges with non-response bias, demographic data collected in the survey suggest our sample was representative of the larger angling community. First, the sex of individuals taking our survey was reported as 21% female and 79% male, which is comparable to nationwide angler sexes of 27% female and 73% male [37]. Age was a harder comparison to make between our study and that of nationwide anglers, primarily because the age intervals we used did not match age intervals in nationwide reporting so no direct comparison could be made. However, the overall shape of the age distribution in our study was similar to the percentage of anglers by age in the US [37], in that both age distributions for both sexes increase from the teenager years to peaking sometime in the 30s, with age intervals in the 30s and older being stable around 20%. Although the comparisons using sex and age do not validate that our study was representative of nationwide anglers, the similarity of our demographics to nationwide demographics provide some evidence that our survey respondents may well represent US anglers.

## Conclusions

US recreational anglers are a large population with varied and dynamic motivations for fishing. Although it might be expected that a pandemic would add challenges and uncertainties to the lives of anglers in a way that could further complicate or diversify behaviors, we found that the impacts of the pandemic were similar across 10 US states, and therefore may reasonably well represent all US anglers. A small overall increase in fishing effort was reported, although access restrictions to fishing locations did vary by state. The increase in fishing effort was more pronounced for anglers with lost work hours or lost jobs with more than half indicating increases in recreational fishing, suggesting that angling is a valuable outlet in challenging times. We also found that one out of three anglers changed their primary reason for fishing; many anglers reported fishing to help with mental stress and social and family bonding as increasingly important during the pandemic.

It is likely that the sustained fishing activity we found during the pandemic was driven by the perceived safety of fishing—the vast majority of anglers we surveyed considered recreational fishing to be a safe activity during a pandemic. US recreational anglers are a large group of stakeholders with economic and ecological impacts on the resources they use. The demonstrated willingness of anglers to keep fishing during a public health crisis suggests the preeminence of the activity and that understanding angler effort, motivation, and resulting behaviors is important for any type of resource management.

## Supporting information

S1 Table List of significant pair-wise comparisons based on a Tukey HSD test. The data underlying these comparisons is shown in Fig 3. Comparisons are also grouped based on the ANOVA model in which they were found (following panels 3A–3C in Fig 3).
(DOCX)

S1 Data COVID fishing data. (CSV)

S1 File Metadata for Coronavirus and recreational angling survey data. (TXT)

S1 Appendix (PDF)
