# Supplementary material for: COVID-19 influences on US recreational angler behavior
Source: PLoS One. 2021 Aug 18;16(8):e0254652. doi: 10.1371/journal.pone.0254652 (PMC8372955; doi:10.1371/journal.pone.0254652)
Supplement: S1 Table — The data underlying these comparisons is shown in Fig 3. Comparisons are also grouped based on the ANOVA model in which they were found (following panels 3A–3C in Fig 3). (DOCX) [file pone.0254652.s001.docx]

S1 Table. List of significant pair-wise comparisons based on a Tukey HSD test. The data underlying these comparisons is shown in Figure 3. Comparisons are also grouped based on the ANOVA model in which they were found (following panels A–C in Fig 3).

| **Fishing trips before the pandemic** | |
| --- | --- |
|  | Mental stress – No effect |
|  | Income loss – Mental stress |
|  | Family – Mental stress |
|  | Job loss – Mental stress |
|  | Job loss – Childcare |
|  | Job loss – Work hours |
|  |  |
| **Fishing trips during the pandemic** | |
|  | Mental stress – No effect |
|  | Job loss – No effect |
|  | Job loss – Physical health |
|  | Work hours – Mental stress |
|  | Job loss – Mental stress |
|  | Job loss – Other |
|  | Job loss – Income loss |
|  | Job loss – Childcare |
|  |  |
| **Difference in fishing trips** | |
|  | Physical health – No effect |
|  | Work hours – No effect |
|  | Mental stress – Physical health |
|  | Income loss – Physical health |
|  | Childcare – Physical health |
|  | Work hours – Physical health |
|  | Job loss – Physical health |
|  | Work hours – Mental stress |
|  | Job loss – Mental stress |
|  | Work hours – Other |
|  | Job loss – Other |
|  | Work hours – Income loss |
|  | Job loss – Income loss |
|  | Work hours – Family |
|  | Job loss – Family |
