# Supplementary material for: COVID-19 influences on US recreational angler behavior
Source: PLoS One. 2021 Aug 18;16(8):e0254652. doi: 10.1371/journal.pone.0254652 (PMC8372955; doi:10.1371/journal.pone.0254652)
Supplement: S1 Appendix — (PDF) [file pone.0254652.s004.pdf]

Survey: Coronavirus and Fisheries

Consent form and FAQs omitted

Q5 Please select the aspects of your life that were/are affected by the coronavirus pandemic. (You may select multiple responses.)

- ☐ I contracted Coronavirus (COVID-19) (1)
- ☐ An immediate family member contracted Coronavirus (COVID-19) (2)
- ☐ My income was reduced (3)
- ☐ My work hours were reduced (independent from income) (4)
- ☐ I lost my job (5)
- ☐ I lost childcare and took care of my kids during the day (6)
- ☐ My physical health declined (7)
- ☐ My level of mental stress increased (8)
- ☐ I was not negatively affected (9)
- ☐ Other (describe) (10) ____

Carry Forward Selected Choices from "Please select the aspects of your life that were/are affected by the coronavirus pandemic. (You may select multiple responses.)"

X→

Q6 Please rank the aspects of your life that you indicated were/are affected from most to least by the coronavirus pandemic
______ I contracted Coronavirus (COVID-19) (1)
______ An immediate family member contracted Coronavirus (COVID-19) (2)
______ My income was reduced (3)
______ My work hours were reduced (independent from income) (4)
______ I lost my job (5)
______ I lost childcare and took care of my kids during the day (6)
______ My physical health declined (7)
______ My level of mental stress increased (8)
______ I was not negatively affected (9)
______ Other (describe) (10)

End of Block: Coronavirus Effects

Start of Block: Fishing Behaviors

Q7 Approximately how often do you purchase a fishing license?

○ I purchase a fishing license most years (1)

○ I do NOT purchase a fishing license most years (2)

○ I am licensed on a longer timeframe (lifetime, senior, etc.) (3)

Q8 If you purchase an annual license, in which season do you ***typically*** purchase it?

○ Winter (December–Febraury) (1)

○ Spring (March–May) (2)

○ Summer (June–August) (3)

○ Fall (September–November) (4)

Q9 Did the Coronavirus pandemic change your fishing license purchasing in 2020?

- ◯ No change (1)
- ◯ The pandemic delayed my purchased (2)
- ◯ The pandemic hastened my purchase (3)
- ◯ I decided not to purchase a license in this time frame (but I typically would) (4)
- ◯ I decided to purchase a license (when I wasn’t planning to purchase one this year) (5)

Q10 If you were licensed to fish in the spring of 2020, did you fish:

- ◯ A lot more than I would in a typical year (1)
- ◯ A little more than I would in a typical year (2)
- ◯ About the same as I would in a typical year (3)
- ◯ A little less than I would in a typical year (4)
- ◯ A lot less or not at all than I would in a typical year (5)

Q11 **Prior** to the Coronavirus pandemic, please select the reasons you typically fished. (You may select multiple responses.)

- ☐ Food (1)
- ☐ Sport or Thrill (2)
- ☐ Nature or being outdoors (3)
- ☐ Social/Family bonding (4)
- ☐ Stress Relief (5)
- ☐ Competition with friends (6)
- ☐ Because I had free time (7)
- ☐ To get away from people (8)
- ☐ Other (9) ____
- ☐ I do not typically fish (10)

Carry Forward Selected Choices from "Prior to the Coronavirus pandemic, please select the reasons you typically fished. (You may select multiple responses.)"

Q12 Please rank the reasons you typically fished **prior** to the Coronavirus pandemic
______ Food (1)
______ Sport or Thrill (2)
______ Nature or being outdoors (3)
______ Social/Family bonding (4)
______ Stress Relief (5)
______ Competition with friends (6)
______ Because I had free time (7)
______ To get away from people (8)
______ Other (9)
______ I do not typically fish (10)

Q13 During the Coronavirus pandemic, please select the reasons you fished. (You may select multiple responses.)

▢ Food (1)

▢ Sport or Thrill (2)

▢ Nature or being outdoors (3)

▢ Social/Family bonding (4)

▢ Stress Relief (5)

▢ Competition with friends (6)

▢ Because I had free time (7)

▢ To get away from people (8)

▢ Other (9) ____

▢ I did not fish during the pandemic (10)

Carry Forward Selected Choices from "During the Coronavirus pandemic, please select the reasons you fished. (You may select multiple responses.)"

Q14 Please rank the reasons you typically fished **during** the Coronavirus pandemic

_______ Food (1)
_______ Sport or Thrill (2)
_______ Nature or being outdoors (3)
_______ Social/Family bonding (4)
_______ Stress Relief (5)
_______ Competition with friends (6)
_______ Because I had free time (7)
_______ To get away from people (8)
_______ Other (9)
_______ I did not fish during the pandemic (10)

Q15 In a typical year (not 2020), about how many fishing trips would you take during the months of March – May?

	0	5	10	15	20	25	30	35	40	45	50
Number of March–May trips in typical year ()											

Q16 During March – May 2020, how many fishing trips did you take?

	0	5	10	15	20	25	30	35	40	45	50
Number of March–May trips in 2020 ()											

Q17 **Prior** to the Coronavirus pandemic, where did you typically fish (select all that apply)?

- ☐ Saltwater: Offshore (1)
- ☐ Saltwater: Inshore or estuarine (2)
- ☐ Freshwater: Ponds (3)
- ☐ Freshwater: Lakes (4)
- ☐ Freshwater: Streams or rivers (5)
- ☐ other (6) ____

Q18 During the Coronavirus pandemic, where did you typically fish (select all that apply)?

- ☐ Saltwater: Offshore (1)
- ☐ Saltwater: Inshore or estuarine (2)
- ☐ Freshwater: Ponds (3)
- ☐ Freshwater: Lakes (4)
- ☐ Freshwater: Streams or rivers (5)
- ☐ other (6)

Q19 Was any of your fishing access changed during the coronavirus pandemic (spring 2020)? For example, were boat ramps closed and unavailable for use?

- ◯ All my fishing access was closed (1)
- ◯ Some of my fishing access was closed (2)
- ◯ None of my fishing access was changed (3)

Q20 If your fishing access was changed, please tell us how it changed. (For example, boat ramps closed or public parks closed)

____

Q21 Whether you fished or not during spring 2020, how safe do you think fishing is?

- ◯ Extremely safe (1)
- ◯ Somewhat safe (2)
- ◯ Unsure (3)
- ◯ Somewhat unsafe (4)
- ◯ Extremely unsafe (5)

End of Block: Fishing Behaviors

Start of Block: Demographics

Q22 What is your age?

- 17 or younger (1)
- 18–20 (2)
- 21–29 (3)
- 30–39 (4)
- 40–49 (5)
- 50–59 (6)
- 60 or older (7)

Q23 What is your gender?

- Male (1)
- Female (2)
- Prefer not to answer (3)

Q24 How much total combined money did all members of your household earn last year?

- Less than $10,000 (1)
- $10,000 - $19,999 (2)
- $20,000 - $29,999 (3)
- $30,000 - $39,999 (4)
- $40,000 - $49,999 (5)
- $50,000 - $59,999 (6)
- $60,000 - $69,999 (7)
- $70,000 - $79,999 (8)
- $80,000 - $89,999 (9)
- $90,000 - $99,999 (10)
- $100,000 - $149,999 (11)
- More than $150,000 (12)

End of Block: Demographics
